# Conditionally Discharged Restricted Patients in a General Adult Community Mental Health Setting

**DOI:** 10.1192/bjo.2025.10464

**Published:** 2025-06-20

**Authors:** Adekunle Adesola, Muhammad Saleem, Marlene Kelbrick, Zakaria Halim, Joel Nkire

**Affiliations:** 1 Northamptonshire Healthcare NHS Foundation Trust, Northampton, United Kingdom; 2 University Hospitals of Derby and Burton NHS Foundation Trust, Derby, United Kingdom

## Abstract

**Aims:** Little is known about the proportion and patient profile of conditionally discharged patients supervised by general adult community mental health teams (CMHTs). In this study we aimed to evaluate the number of patients and their demographic, clinical and risk profile, and current practice in terms of supervision and structures.

**Methods:** We conducted a retrospective case note service evaluation of all conditionally discharged patients within a typical NHS Trust’s CMHTs.

**Results:** A third of all conditionally discharged patients within the Trust were supervised under the care of general adult community teams. The majority of patients were older, male, unemployed with schizophrenia and related disorder diagnoses. Main index offences were serious violence to others with use of weapons.

**Conclusion:** Conditionally discharged patients represent a low volume, high risk population. Supervision in the community is time and resource intensive. There is a need for NHS Trusts to ensure adequate support and structures, supervision, training, and joint working opportunity with forensic services to ensure safe quality care.

